# Mycorrhizal network: a bidirectional pathway between green-leaved terrestrial orchids and pine trees

**DOI:** 10.3389/fpls.2025.1620153

**Published:** 2025-10-28

**Authors:** Jianxin Chen, Xiang Ma, Fengjinglin Wu, Na Qiao, Xingliang Xu, Jianrong Wu

**Affiliations:** ^1^ Key Laboratory of Forest Disaster Warning and Control of Yunnan Province, College of Forestry, Southwest Forestry University, Kunming, China; ^2^ School of Life Science and Technology, University of Electronic Science and Technology of China, Chengdu, China; ^3^ Key Laboratory of Ecosystem Network Observation and Modelling, Institute of Geographic Sciences and Natural Resources Research, Chinese Academy of Sciences, Beijing, China; ^4^ Key Laboratory of National Forestry and Grassland Administration on Biodiversity Conservation in Southwest China, College of Forestry, Southwest Forestry University, Kunming, China

**Keywords:** bidirectional pathway, mycorrhizal network, orchid mycorrhiza, pine seedlings, ^13^C and ^15^N dual labelling

## Abstract

**Introduction:**

Increasing evidence demonstrates that plant roots can be connected via mycorrhizal networks. Such networks in roots play key roles in plant physiology and ecosystem functioning, but it remains debated whether bidirectional transfers of resources can occur simultaneously inside the network.

**Methods:**

We constructed a compartmented system to test for bidirectional carbon (C) and nitrogen (N) transfer between three terrestrial orchids (*Cymbidium goeringii*, *C. goeringii* var. *serratum*, and *C. faberi*) and Pinus yunnanensis seedlings, which were linked via a common *Ceratobasidium* sp. A ^13^C and ^15^N dual labelling approach was employed to trace the simultaneous movement of both elements.

**Results:**

A unidirectional transfer of C and N was observed between *C. goeringii* and pine seedlings. In contrast, simultaneous bidirectional transfer of both elements occurred between the other two orchid species and pine seedlings; 1.0–3.7% of assimilated C and 0.20–12.2% of acquired N were transferred through the network. The net C transfer was consistently directed from pine seedlings to the orchids. Nitrogen transfer exhibited three distinct, species-specific patterns: (i) unidirectional from *C. goeringii* to pine, (ii) bidirectional with no net transfer between *C. faberi* and pine, and (iii) bidirectional with a net transfer from *C. goeringii* var. *serratum* to pine.

**Discussion:**

The divergent transfer patterns among orchid species demonstrate that mycorrhizal networks function as dynamic, species-specific pathways for resource transfer. This specificity may significantly influence orchid recruitment and nutrient dynamics in forest understories, suggesting that the role of common mycorrhizal networks is more complex than previously recognized.

## 1 Introduction

More than 80% of all terrestrial plant species are symbiotically associated with mycorrhizal fungi (Weng et al., 2022). Such mycorrhizal symbioses lead to symbiotic exchange of resources between fungi and their host plants, *i.e.* the fungal partner supplies host plants with limiting nutrients in return for photosynthetic assimilates (Newman, 1988; Smith and Read, 2008). Moreover, mycorrhizal networks often develop between neighboring plants via hyphal connections in a variety of terrestrial ecosystems (Robinson and Fitter, 1999; Simard and Durall, 2004). An increasing number of studies confirmed such links between different plant species (Selosse et al., 2006) and demonstrated that mycorrhizal networks facilitate interplant resource transfer (Teste et al., 2009), contribute to formation and maintenance of soil structure, and support plant diversity as well as plant defense (van der Heijden et al., 2015). Nevertheless, this view has been challenged. Critical reviews argue that evidence for substantial net resource transfer is limited, and that observed patterns could be explained by alternative pathways or fungal retention of carbon, thus questioning the prevalence and importance of common mycorrhizal networks mediated resource sharing in plant communities (Karst et al., 2023; Audisio et al., 2024).

However, their function in interplant resource transfer remains a matter of debate, particularly whether such transfer is directional (Robinson and Fitter, 1999; Jakobsen and Hammer, 2015; Simard et al., 2015). A large number of studies have suggested a directional transfer of carbon (Francis and Read, 1984; Wu et al., 2001; Gebauer and Meyer, 2003; Julou et al., 2005; Cameron et al., 2006, 2008; Newman, 2008; Bougoure et al., 2010; Hynson et al., 2013; Klein et al., 2016), nitrogen (Haystead et al., 1988; Rogers et al., 2001; Govindarajulu et al., 2005; Julou et al., 2005; Cameron et al., 2006; He et al., 2006; Bougoure et al., 2010; Hynson et al., 2013), and phosphorus (Chiariello et al., 1982; Whittingham and Read, 1982; Ritz and Newman, 1984; Wilson et al., 2006) between plants through mycorrhizal networks. Based on the source-sink theory (Kytöviita et al., 2003) and the biological market theory (Noë and Hammerstein, 1995; Schwart and Hoeksema, 1998), resource transfer should be directed to the sink or the side with greater demand. However, the dynamics of water transport (Querejeta et al., 2003; Stuefer et al., 2004) and signal transfer (Johnson and Gilbert, 2015) within mycorrhizal networks suggest that the networks could be a bidirectional pathway for resources. Several lines of evidence have suggested a bidirectional pathway for carbon transfer between plants through arbuscular mycorrhizal (Grime et al., 1987) or ectomycorrhizal (Klein et al., 2016) networks. Simard and Durall (2004) further suggested several possible pathways for carbon transfer between two plants through ectomycorrhizal networks. However, these results were questioned after numerous studies demonstrated that carbon is mostly retained in roots or fungal tissues and is not further transferred from roots to shoots for plant utilization (Robinson and Fitter, 1999; Pfeffer et al., 2004; Jakobsen and Hammer, 2015). Thus, the core issues for untangling the roles of mycorrhizal networks and their ecological significance are whether these networks serve as bidirectional conduits for transfer of different resources between connected plants and to unveil the magnitude of transferred resources (Robinson and Fitter, 1999; Jakobsen and Hammer, 2015; Simard et al., 2012, 2015; van der Heijden, 2016). Numerous studies have investigated bidirectional transfer of carbon or nitrogen separately (Jakobsen and Hammer, 2015; Simard et al., 2015). Recent studies have shown that trees retain carbon in their roots (Douglas-fir recipients shared on average one ECM species with donors and showed lower 13C enrichment than beech recipients, which shared three species on average) (Audisio et al., 2024), and that global vegetation allocates more carbon to roots than to leaves (Duanmu et al., 2025). However, few studies have examined whether bidirectional transfer of two resources (*e.g.* carbon and nitrogen) occurs simultaneously within mycorrhizal networks between plants while concurrently assessing the quantitative significance of such transfers.

Previous studies mainly focused on mycorrhizal networks consisting of arbuscular mycorrhizal fungi (Jakobsen and Hammer, 2015) or ectomycorrhizal fungi (Simard et al., 2015), while the number of studies on orchid mycorrhizal fungi have increased in the last two decades. Using natural stable isotope abundance approach, several studies suggested that the net tripartite matter flux could occur between trees, fungi, and orchids (Gebauer and Meyer, 2003; Bidartondo et al., 2004; Julou et al., 2005; Hynson et al., 2013), although this approach cannot evaluate bidirectional transfer of carbon and nitrogen. Further studies used ^13^C, ^14^C, or ^15^N labelling and demonstrated a bidirectional transfer of carbon between a green orchid and its fungal symbiont and a fungus-dependent pathway for organic N acquisition by orchids (Cameron et al., 2006, 2008; Bougoure et al., 2010). Using compartmentalized microcosms together with a ^13^C labelling approach, Bougoure et al. (2010) confirmed that carbon can be transferred to the understory orchid by a shrub via a common mycorrhizal fungus. However, it remains unclear whether bidirectional transfer of carbon and nitrogen occurs simultaneously within mycorrhizal networks between pine and green orchids in subtropical and tropical forests. In such forests, various fungi forming mycorrhizas with terrestrial green orchids typically live as saprotrophs in the soil or form endophytic/ectomycorrhizal associations with neighboring trees (Bidartondo et al., 2004; Dearnaley et al., 2013).

To enable the investigation of bidirectional resource transfer within mycorrhizal networks, a gross simplification of real-world complexity has been suggested: two plant species are linked by one fungus (Whitfield, 2007). In this study, we set-up an experiment with compartmented microcosms to establish mycorrhizal symbiosis between terrestrial green orchids and a pine tree. A native pine tree species, *Pinus yunnanensis*, and three orchid taxa, *Cymbidium goeringii*, *C*. *goeringii* var. *serratum*, and *C*. *faberi*, were used. These plant species were selected for two reasons. First, they are often observed in subtropical forests in the Yunnan Province of China, and all three orchid taxa grow frequently in the forest understory in the region. Second, we obtained a fungus from *C*. *goeringii* rhizomes in a previous study (Wu et al., 2010); this fungus forms easily mycorrhizal associations with orchids from the genus *Cymbidium*. Such *Cymbidium* orchids are mixotrophic (Motomura et al., 2010), and when they grow together with *P*. *yunnanensis* seedlings under the same light and soil resource conditions, the orchid and pine seedlings may have distinct resource requirements. Here, we hypothesize the following: (1) A bidirectional transfer of carbon occurs between orchids and pine seedlings through the mycorrhizal network, but with a net carbon transfer from autotrophic pines to mixotrophic orchids; (2) Nitrogen can be transferred bidirectionally between orchids and pine seedlings through the mycorrhizal network, with a net transfer from orchids to pine because mixotrophic orchids could acquire more nitrogen via fungal hypha; and (3) Orchid identity could affect the net tripartite matter fluxes. Because the fungus was isolated from *C*. *goeringii* rhizomes, we hypothesized that its inoculation would enhance carbon and nitrogen transfer between *C*. *goeringii* and pine seedlings compared to the other two *Cymbidium* orchid taxa. To test these hypotheses, the ^13^C and ^15^N dual labelling method was used in this study.

## 2 Materials and methods

### 2.1 Fungal strain isolation

To obtain natural mycorrhizal fungal strains, *C*. *goeringii* rhizomes were collected from a coniferous forest of *P*. *yunnanensis* and from a mixed evergreen broad-leaved forest of *Quercus acuta* and *P*. *yunnanensis* in Jindian Conservation Area (25°04'N, 102°45'E, 1780 m above sea level), located in Kunming City, Yunnan Province. The soils in Jindian Park are clay-loam with high iron/aluminum oxide content, Slightly acidic to neutral (pH 5.5–6.8). The annual mean temperature ranged from 11.2 to 13.8 °C and the annual mean precipitation varied from 980 to 1156 mm. Mycorrhizal fungi were isolated from the collected rhizomes according to the protocol described by Wu et al. (2010). Briefly, the rhizome surface was sterilized with 70% ethanol for 1 min followed by 1% sodium hypochlorite for 1 min. Segments (~5 mm) were crushed in sterile water to release pelotons, which were dispersed in Modified Melin-Norkrans (MMN) agar medium and incubated at 25 °C in the dark. Emerging fungal colonies were subcultured and identified by phylogenetic analysis of the internal transcribed spacer (ITS) region of the ribosomal DNA (Saitou and Nei, 1987). The fungal strain used in this study, designated as CL111KM, has been deposited in the Culture Collection of Southwest Forestry University.

### 2.2 Non-mycorrhizal seedling

Ripe seeds of three terrestrial orchid species (*C*. *goeringii*, *C*. *goeringii* var. *serratum*, and *C*. *faberi*) were sterilized and germinated as non-mycorrhizal seedlings on agar. Orchid seedlings were grown at 25 °C with a photoperiod of light: dark (12 h:12 h) cycle under fluorescent lamps (800 μmol m^-2^ s^-1^). After growing for 12 months, the seedlings with five leaves and a height of 8 cm were transplanted to a sterilized substrate that contained dried and washed mosses (collected from the forest floor and they were washed and sterilized before they were mixed to constitute the substrate for plants), vermiculite, and sand in a ratio of 1:1:1 (v/v/v). These seedlings were allowed to grow for an additional month to adapt to soil environments in the glasshouse. To obtain non-mycorrhizal seedlings of *P*. *yunnanensis*, pine seeds were first sterilized and then germinated in a substrate that was sterilized by dry heat at 160 °C for 72 h to eliminate any microorganisms. The substrate consisted of soil from organic and mineral horizons (1:2, v/v), which were collected from the *P*. *yunnanensis* forest. After growing for 12 months, pine seedlings reached a height of 10 cm and contained 3–5 branches. To prevent colonization by airborne microorganisms in the glasshouse and any dripping of labelling solution onto the soil surface, the soil surface of all seedlings was covered with a thin plastic film.

### 2.3 Establishment of mycorrhizal associations

**Figure 1:**
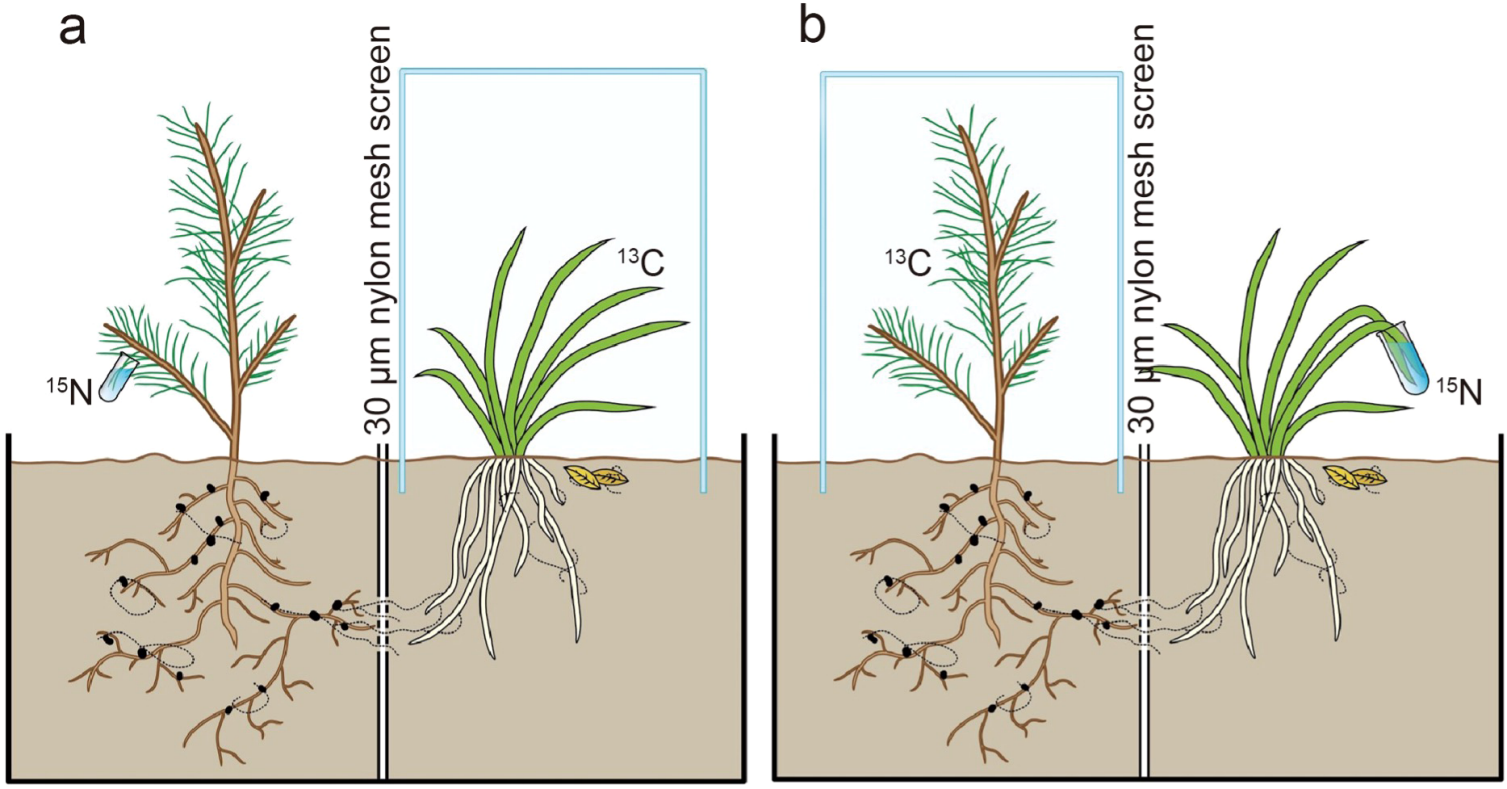
Double-split boxes were used to establish mycorrhizal symbioses between orchid and pine seedlings. Simultaneous ^13^C and ^15^N dual labelling commenced after the symbiosis had been established for 12 months: **(a)** Simultaneous ^13^C labelling of orchid seedlings and ^15^N labelling of pine; **(b)** Simultaneous ^15^N labelling of orchid seedlings and ^13^C labelling of pine.

Compartmented microcosms were used to establish mycorrhizal associations between a fungus, an orchid, and a pine tree. Each compartmented microcosm consisted of two boxes (30 × 30 cm and 30 cm height). A small drainage hole at the center of each microcosm bottom allowed excess water to seep out after watering, and a 0.5 cm space between the two boxes prevented solution flow between the boxes. A window (10 × 10 cm) was cut out at the center of the side facing the other box and covered with a 30 μm nylon mesh screen to prevent roots but allow fungal hyphae to penetrate into the adjacent box (**Figures 1a, b**). Around the windows, foil was used to keep moisture high between the two boxes and hinder hyphal desiccation. When healthy non-mycorrhizal seedlings were ready, they were transplanted into these microcosms, which contained humus and mineral soil in a ratio of 1:1 (v/v); this mixture was previously sterilized by dry heat at 160 °C for 72 h to eliminate any microorganisms from the substrate. The transplanted seedlings were grown in a glasshouse at 25 °C, 70-80% air humidity, and with a natural light-dark cycle typical of Kunming City. After acclimation for 30 d, the orchid seedlings were inoculated with the fungal strain CL111KM. The inoculum was prepared by growing the fungus in liquid MMN medium for 4 weeks at 25 °C. The mycelium was homogenized and adjusted to a concentration of 10^4^ hyphal fragments per mL. Each orchid seedling received 20 mL of this suspension, applied near the root zone. Additionally, Quercus acutissima leaves colonized by the fungal mycelia were buried 1 cm away from the Cymbidium seedlings (Wu et al., 2013). The inoculated seedlings were grown for 12 months to form mycorrhizal networks between orchids and pine seedlings. During the plant growth period in the glasshouse, these microcosms were put on the benches with the distance of approximately 1.5 cm between the microcosms and the benches to prevent any cross contamination between the pots. Moreover, since the roots did not extend out of the pots and therefore could not take up nitrogen or carbon from leachates of other pots, hyphal growth between microcosm bottoms was thus hindered by the space between pot bottoms and the benches (no visible hypha coming out of the pots through drainage holes). Experimental setup included 15 total plant associations.

Five fresh root tips (0.5 cm size) were randomly selected from one plant individual of each treatment and fixed in formalin–acetic acid–alcohol. The fixed samples were embedded into paraffin, cut into sections, and stained with hematoxylin and eosin. The stained paraffin sections were observed under an optical microscope (Nikon-YS100; Tokyo, Japan) to count the number of infected root segments, which was then used to calculate mycorrhizal colonization rate (Phillip and Hayman, 1970; Stefani et al., 2010).

### 2.4 Isotope labelling

After the mycorrhizal associations were formed between the orchids and pine seedlings, the plants were labelled symbiotically with ^13^C and ^15^N, *i.e.* orchid seedlings were labelled with ^13^CO_2_ and pine seedlings in the same compartmented microcosm were simultaneously labelled with ^15^N (**Figure 1a**), and vice versa (**Figure 1b**). At the beginning of the ^13^C labelling, the ^15^N labelling was performed simultaneously by soaking green leaves (two leaves for orchid seedlings and three clusters of leaves for pine seedlings) in a 50 mM ^15^NH_4_^15^NO_3_ solution (98 atom% ^15^N). Such foliar nitrogen fertilization/foliar nitrogen application is a common procedure in horticulture and has been adopted multiple times to apply tracers to intact plant-soil systems without negative but instead rather positive effects on plants *e.g.* (Tomaszewski and Sievering, 2007). Plants take up nitrogen from foliar applications in natural settings *e.g.* forests, grasslands and croplands (McNeill et al., 1997; Gaige et al., 2007; Sievering et al,. 2007; Wichern et al., 2008; Adriaenssens et al., 2011; 2012) and plant-mycorrhiza systems (Høgh-Jensen and Schjoerring, 2000; He et al., 2006; Teste et al., 2015), and the concentration applied is in the normal range usually applied as urea or ammonium nitrate (Chalk et al., 2014). In this study, only a small section of each leaf was soaked in ^15^N labelling solution to avoid damage to the leaves (He et al., 2006). During the labelling care was taken to avoid spillage of the labelling solution onto the soil surface and to prevent N leaching from leaves. The ^13^C labelling was conducted only during the first 20 h in a closed glass box (3 L) by adding 5% H_2_SO_4_ to Ba^13^CO_3_ (99 atom% ^13^C) to produce ^13^CO_2_. Its concentration was maintained at about 410 ppm through controlled addition of 10% H_2_SO_4_ every 4 h. After ^13^C labelling, microcosms were removed from the closed glass boxes and a fan was installed close to each experimental unit to remove soil ^13^CO_2_ and avoid its re-assimilation by unlabeled seedlings. The ^15^N labelling was allowed to perform for an additional 52 h after the ^13^CO_2_ labelling. Reference microcosms, which were not amended with ^13^C and ^15^N tracers, were used as controls. Four replicates per treatment were established. Plants were harvested destructively 72 h after the start of the ^15^N labelling. The aboveground parts and roots were retrieved, washed, and dried in an oven at 75 °C for 48 h. Dried plant materials were weighed and ground to a fine powder with a ball mill (MM200, Retsch, Haan, Germany) for stable isotope analysis.

### 2.5 Isotope analysis

Aliquots of plant samples were weighed into tin capsules for analyzing C%, N%, as well as ^13^C/^12^C and ^15^N/^14^N ratios by continuous-flow gas isotope ratio mass spectrometry (CF-IRMS), which was coupled to an elemental analyzer (EA 1110, CE Instruments, Milan, Italy), a ConFlo III device (Finnigan MAT, Bremen, Germany), and a gas isotope ratio mass spectrometer (MAT253, Finnigan MAT). Isotopic reference materials calibrated to atmospheric N_2_ and Vienna-Pee Dee Belemnite international standards were used between samples. Standard deviation of repeated measurements for laboratory standards was ±0.15‰ for carbon and nitrogen isotopes in delta notation.

### 2.6 Calculations and statistics

Atom% ^15^N excess (APE ^15^N) was calculated as the atom% ^15^N difference between the labelled seedlings (atom%_labeled_) and those from the control microcosms (atom%_control_) of the same plant species; APE ^13^C was determined analogously as described by Equation 1.


(1) $${\text{APE}}^{15}{\text{N or}}^{13}\text{C}=\text{atom}{\%}_{\text{labelled}}–\text{atom}{\%}_{\text{control}}$$

Assimilation of ^15^N by foliar ^15^N-labelled plants (^15^N_assimilated_, μg) was calculated by multiplying APE ^15^N by nitrogen content (N%) and biomass (g) (Equation 2); the amount of ^13^C fixed by the ^13^CO2-labelled plants (^15^C_fixed_, μg) was calculated by multiplying APE ^13^C by carbon content (C%) and biomass (g) (Equation 3).


(2) Nassimilated15(μg)=APE15N×N%×biomass×106

(3) Cfixed13(μg)=APE13C×C%×biomass×106

The amount of ^15^N transferred (^15^N_transferred_, μg) from the labelled to the receiving plant through the mycorrhizal network was calculated by multiplying APE ^15^N of the receiving plant (APE ^15^N_RE_) by its nitrogen content (N%) and dry biomass (g) (Equation 4); the amount of ^13^C transferred (13C_transferred_, μg) from the labelled to the receiving plant through the mycorrhizal network was calculated by multiplying APE ^13^C of the receiving plant (APE ^13^C_RE_) by its carbon content (C%) and dry biomass (g) (Equation 5).


(4) Ntransferred15(μg)=APE15NRE×N%×biomass×106

(5) Ctransferred13(μg)=APE13CRE×C%×biomass×106

The percent of assimilated ^15^N (^15^NP_TR_) or fixed ^13^C (^13^CP_TR_) from the labelled to the receiving plant through the mycorrhizal network was calculated by dividing assimilated ^15^N or fixed ^13^C by the sum of assimilated ^15^N or fixed ^13^C and transferred ^15^N or ^13^C and multiplying by 100 (see Equations 6 and 7, respectively).


(6) NPTR15(%)=[15Ntransferred/(Ntransferred15+15Nassimilated)]×100

(7) CPTR13(%)=[13Ctransferred/(Ctransferred13+13Cassimilated)]×100

The standard errors of the means were used as a measure of variability. Prior to analysis, the normality of distributions was verified using Shapiro-Wilk tests, and homogeneity of variance was confirmed via Levene’s test. Differences among treatments were assessed using one-way ANOVA in SPSS 22.0 (IBM Corp., USA), followed by Tukey’s HSD *post hoc* test for multiple comparisons (α = 0.05). Statistical significance was set at *P* < 0.05.

## 3 Results

### 3.1 Mycorrhizal association

**Figure 2:**
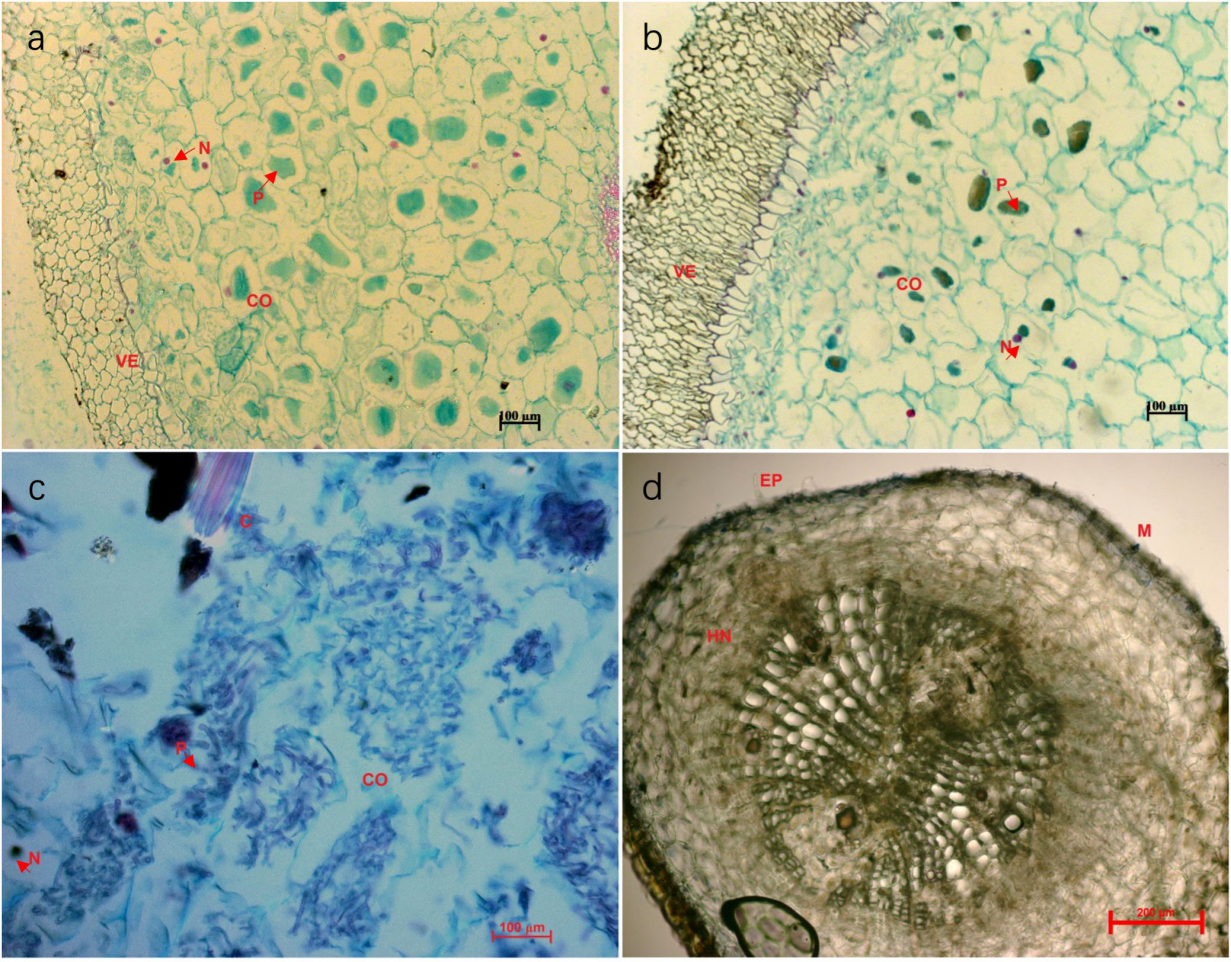
Microstructure of the mycorrhizas of *Cymbidium goeringii* **(a)**, *C. goeringii* var. *serratum* **(b)**, *C. faberi* **(c)**, and *Pinus yunnanensis* **(d)** 12 months after fungal inoculation. The letters in the images indicate root structures: CO, cortex; N, nucleus; P, peloton; VE, velamen; C, needle-shaped crystal; M, Mantle; HN, Hartig net; PI, pith. Red arrows point to the mantle and Hartig net.

Mycorrhizal symbiosis was formed between the terrestrial green-leaved *Cymbidium* orchids and pine seedlings 12 months after fungal inoculation. Root microstructure of the green *Cymbidium* orchids clearly demonstrated formation of orchid mycorrhizas (**Figures 2a, b**). With the establishment of orchid mycorrhizas and extensive fungal growth, the fungi gradually colonized the roots of *P*. *yunnanensis* seedlings and developed ectomycorrhizas (**Figures 2c, d**). Microscopic analysis of stained root sections confirmed that all sampled root segments (n=50 per plant) exhibited intracellular fungal colonization (e.g., pelotons, hyphae) (**Figures 2a, b**), indicating 100% colonization frequency.

**Figure 3:**
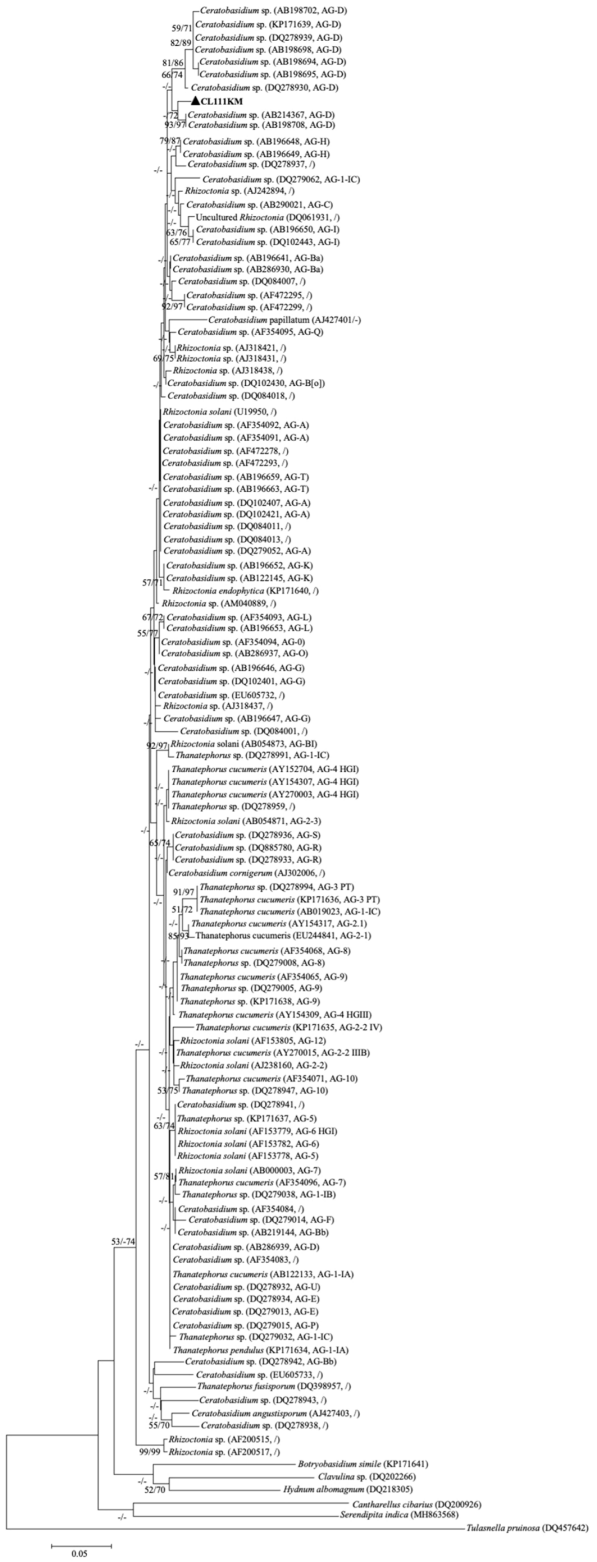
Maximum likelihood and Maximum Parsimony phylogenetic tree showing the relationship between mycorrhizal fungi isolated from *Cymbidium goeringii* and related fungi in Ceratobasidiaceae based on the ITS region of nuclear rDNA. Branches are labelled with maximum likelihood bootstrap higher than 70% and parsimony bootstrap proportions higher than 50% respectively (1,000 replicates). Accession numbers from the DDBJ/EMBL/GenBank nucleotide database are given for all sequences. CL111KM show fungal DNA isolated from ectomycorrhiza with *Pinus yunnanensis*.

The phylogenetic analysis confirms our fungal strain CL111KM clusters robustly with known *Ceratobasidium* sp. (**Figure 3**), particularly those forming ectomycorrhizal associations (Veldre et al., 2013). This placement supports *Rhizoctonia*-like phylogenetic placement among ectomycorrhizal-forming *Ceratobasidium* species.

### 3.2 Biomass

**Figure 4:**
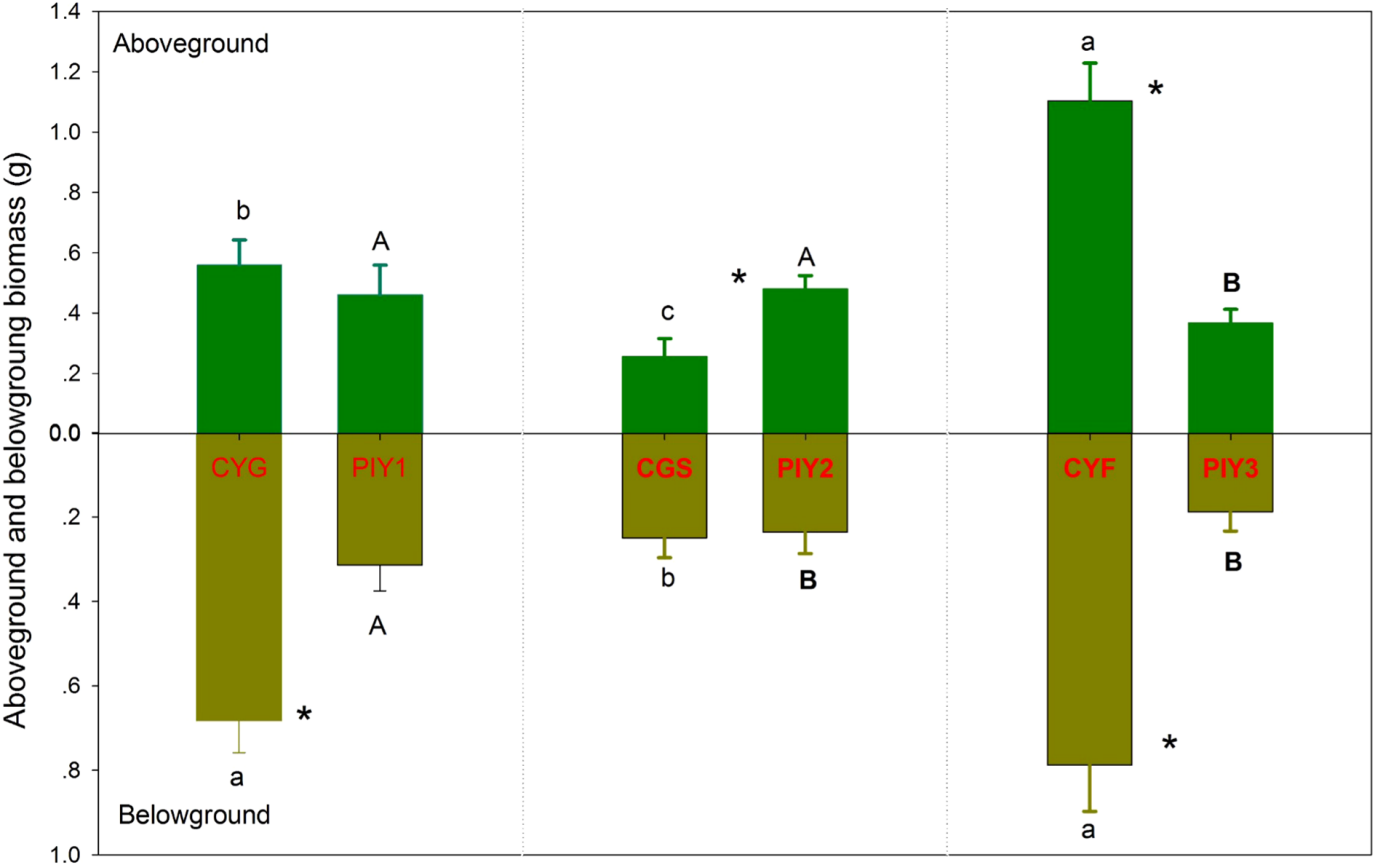
Dry biomass of orchid and pine seedlings in each treatment 12 months after fungal inoculation. The means ± SE of 12 replicates are presented. Different lowercase and uppercase letters indicate significant difference in carbon assimilation and nitrogen acquisition, respectively, within the same plant tissues (leaves *vs*. roots) between orchid and pine seedlings, and “*” indicate significant difference in biomass between orchid and pine seedlings in the same plant tissue at P = 0.05 level (compared of the aboveground and belowground parts separately). PIY, *Pinus yunnanensis*; CYG, *Cymbidium goeringii*; CGS, C. *goeringii* var. *serratum*; CYF, C. *faberi*.

Among the three orchids, *C*. *faberi* showed the highest biomass, while *C*. *goeringii* var. *serratum* had the lowest biomass (**Figure 4**). The biomass of *P*. *yunnanensis* seedlings that were combined with *C*. *goeringii* or *C*. *goeringii* var. *serratum* was similar and higher than the biomass of seedlings combined with *C*. *faberi*. Among the three combinations, the total biomass of *P*. *yunnanensis* seedlings was lower than that of orchids when they were connected with *C*. *goeringii* or *C*. *faberi* but higher when combined with *C*. *goeringii* var. *serratum* (**Figure 4**).

### 3.3 Carbon and nitrogen contents

**Figure 5:**
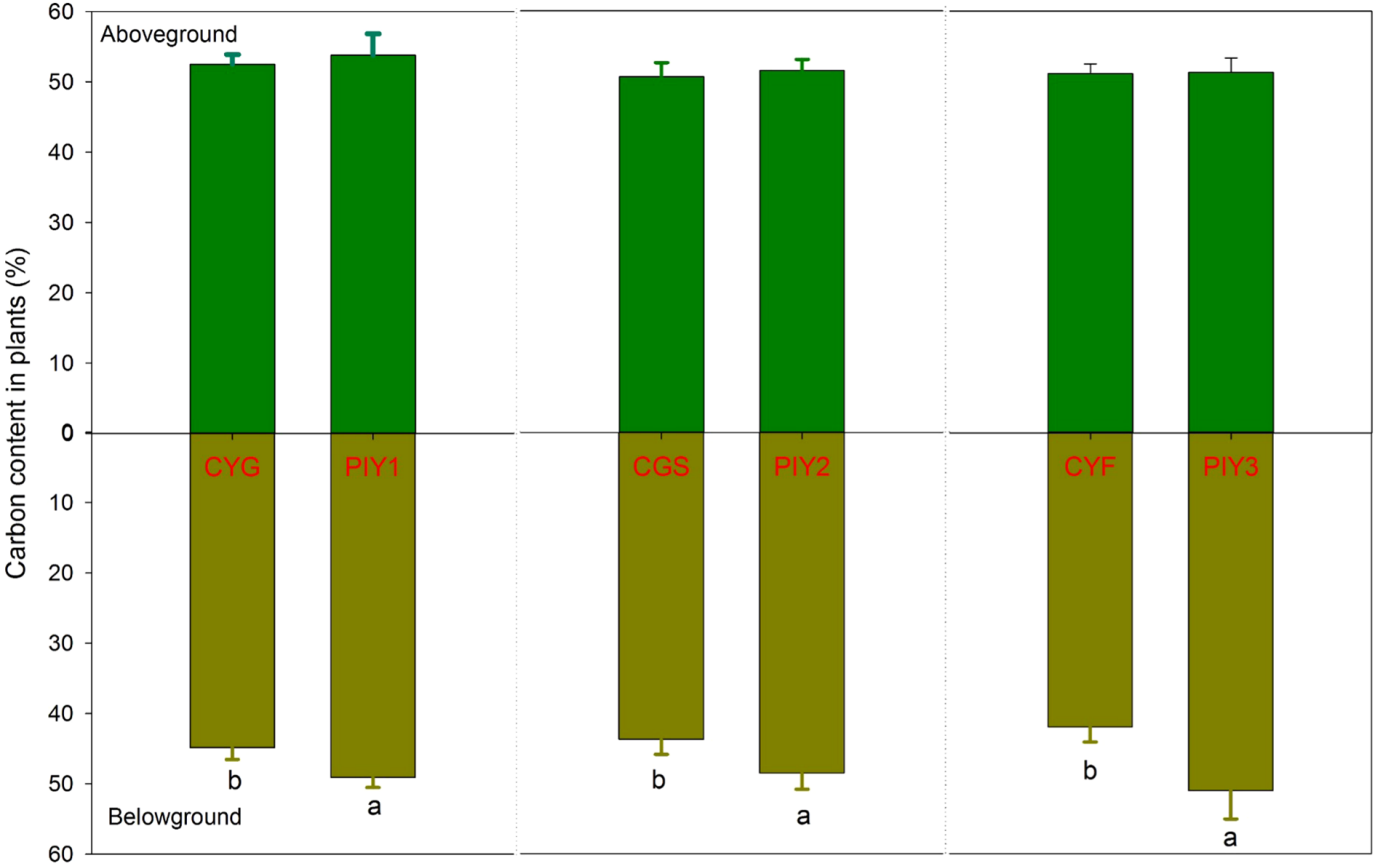
Carbon content of orchid and pine seedlings in each treatment. 12 months after fungal inoculation. The means ± SE of 12 replicates are presented. Different lowercase and uppercase letters indicate significant difference in carbon assimilation and nitrogen acquisition, respectively, within the same plant tissues (leaves *vs*. roots) between orchid and pine seedlings at P = 0.05 level. PIY, *Pinus yunnanensis*; CYG, *Cymbidium goeringii*; CGS, C. *goeringii* var. *serratum*; CYF, C. *faberi*.

**Figure 6:**
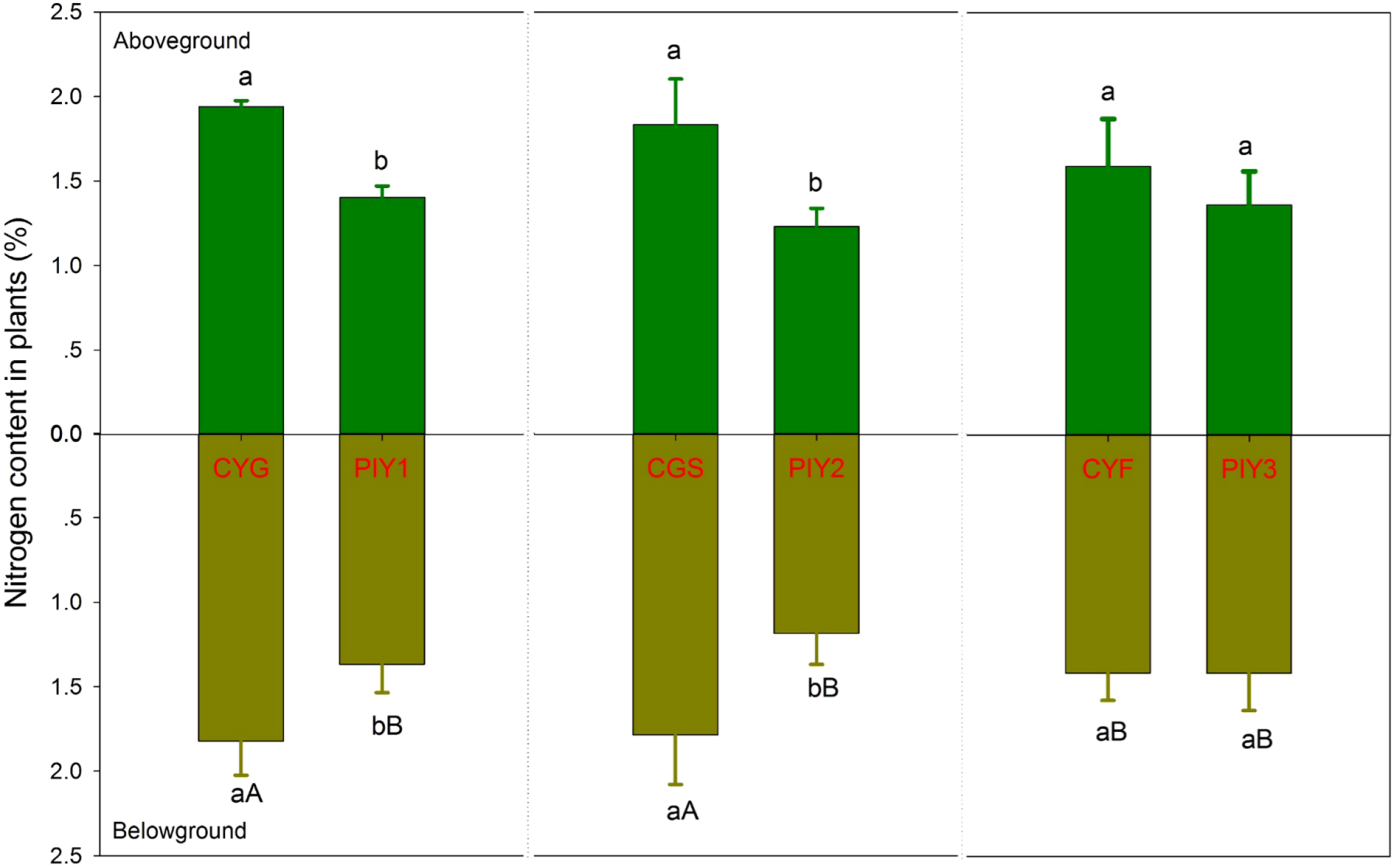
Nitrogen content of orchid and pine seedlings in each treatment 12 months after fungal inoculation. The means ± SE of 12 replicates are presented. Different lowercase and uppercase letters indicate significant difference in carbon assimilation and nitrogen acquisition, respectively, within the same plant tissues (leaves *vs*. roots) between orchid and pine at P = 0.05 level (compared of the belowground parts separately). PIY, *Pinus yunnanensis*; CYG, *Cymbidium goeringii*; CGS, C. *goeringii* var. *serratum*; CYF, C. *faberi*.

In all three combinations, *P*. *yunnanensis* and orchid seedlings had similar carbon content in the aboveground tissues, while *P*. *yunnanensis* demonstrated significantly higher carbon content in the belowground parts than orchids (**Figure 5**). In contrast, the nitrogen content in the seedlings exhibited distinct patterns. When *P*. *yunnanensis* seedlings were combined with *C*. *goeringii* or *C*. *goeringii* var. *serratum*, orchid seedlings showed significantly higher nitrogen content in both the above- and below-ground parts compared to *P*. *yunnanensis* seedlings (**Figure 6**). Neither pine nor orchid seedlings showed significant differences in the nitrogen content of their aboveground parts. By comparison, *C*. *goeringii* var. *serratum* and *C*. *goeringii* showed significantly higher nitrogen content of belowground parts than *C*. *faberi* did (**Figure 6**).

### 3.4 Carbon and nitrogen assimilation and transfer

**Figure 7:**
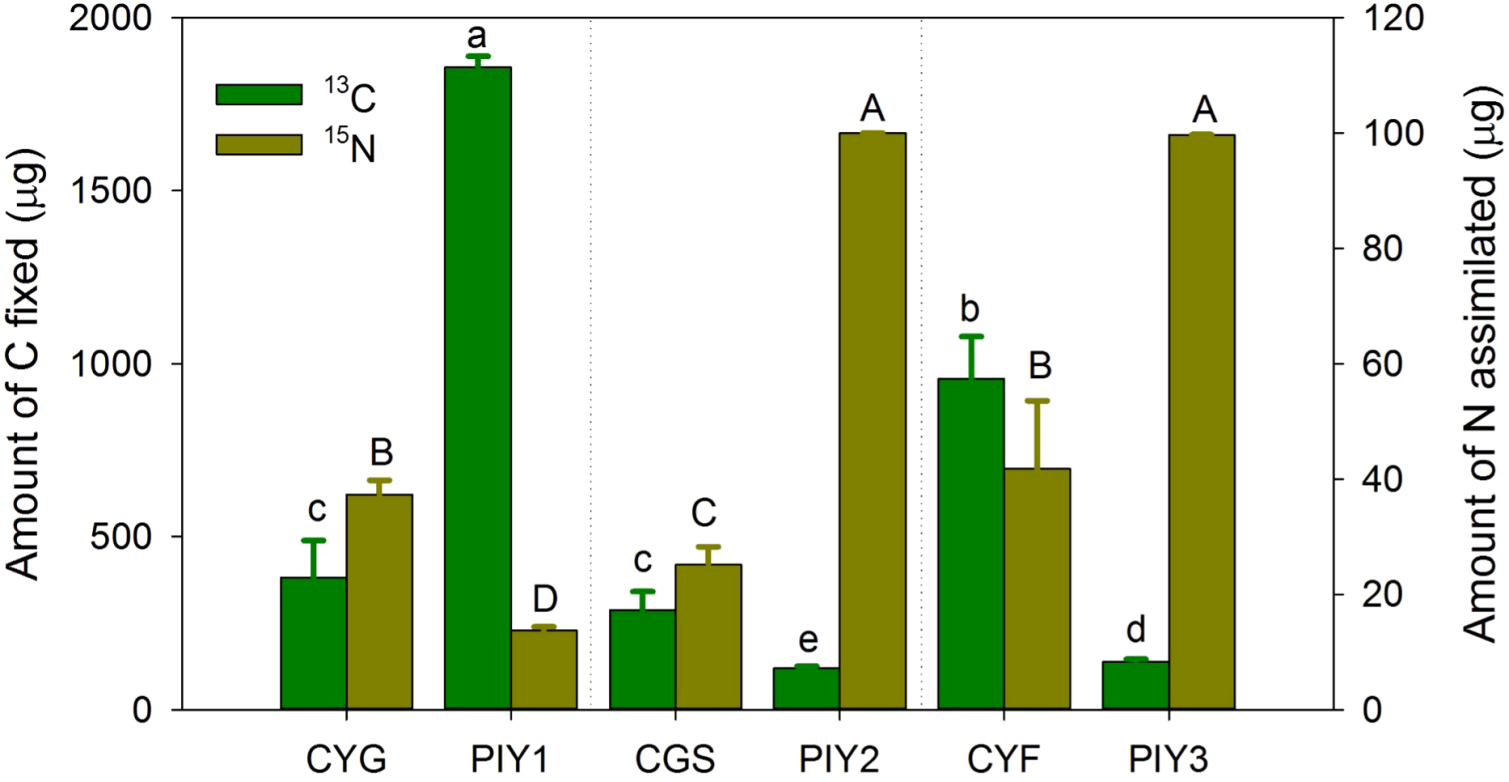
The amount of carbon fixed and nitrogen assimilated by plants. The data show only ^13^C fixation in ^13^CO_2_-labelled plants and ^15^N assimilation in foliar ^15^N-labelled plants, not the receiving plants in the microcosms. The means ± SE of 4 replicates are presented. Different lowercase and uppercase letters indicate significant difference in carbon assimilation and nitrogen acquisition, respectively, between orchid and pine seedlings at P = 0.05 level (compared of the aboveground and belowground parts separately). PIY, *Pinus yunnanensis*; CYG, *Cymbidium goeringii*; CGS, C. *goeringii* var. *serratum*; CYF, C. *faberi*.

**Figure 8:**
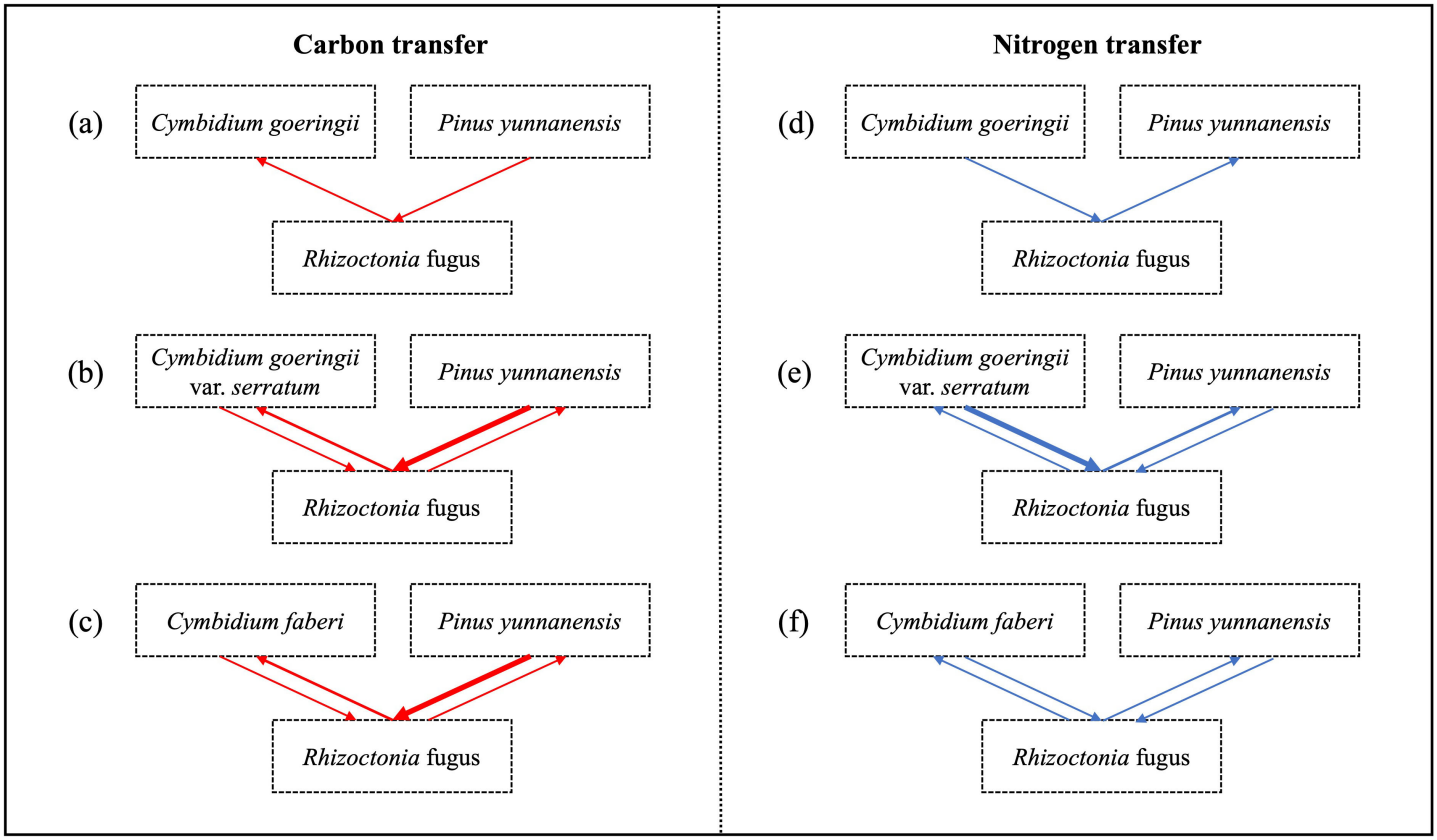
Patterns of carbon and nitrogen transfers between three terrestrial *Cymbidium* orchid taxa and *Pinus yunnanensis* seedlings. **(a-c)** Carbon transfer in the systems with *C. goeringii* **(a)**, *C. goeringii* var. *serratum* **(b)**, and *C. faberi* **(c)**. Red arrows indicate the direction of net carbon transfer. **(d-f)** Nitrogen transfer in the systems with *C. goeringii* **(d)**, *C. goeringii* var. *serratum* **(e)**, and *C. faberi* **(f)**. Blue arrows indicate the direction of net nitrogen transfer. The common mycorrhizal fungus Ceratobasidium sp. is represented in the center of each diagram.

Among the three orchids, *C*. *faberi* assimilated the highest amounts of carbon and nitrogen, while *C*. *goeringii* var. *serratum* fixed the lowest amount of carbon (**Figure 7**). When *P*. *yunnanensis* was connected with *C*. *goeringii*, it fixed the most carbon but assimilated the least nitrogen. In contrast, it fixed the most nitrogen when combined with the other two orchids (**Figure 7**). Only a unidirectional transfer of carbon and nitrogen was observed between *C*. *goeringii* and *P*. *yunnanensis* seedlings. Approximately 1.4 ± 0.1% of carbon photosynthetically fixed by pine seedlings was transferred to green orchid seedlings, while 0.30 ± 0.02% of nitrogen acquired by orchid leaves was transferred to pine seedlings (**Supplementary Figure S1**). In contrast, simultaneous bidirectional transfer of carbon and nitrogen was found between the other two *Cymbidium* orchids (*C*. *goeringii* var. *serratum* and *C*. *faberi*) and *P*. *yunnanensis* seedlings. *C*. *goeringii* var. *serratum* transferred approximately 2.7 ± 0.2% of fixed carbon and 9.0 ± 1.3% of acquired nitrogen to their neighboring pine seedlings, while *P*. *yunnanensis* transferred about 1.4 ± 0.2% of gained carbon and 0.25 ± 0.02% of acquired nitrogen to *C*. *goeringii* var. *serratum* seedlings (**Supplementary Figure S1**). The amounts of carbon and nitrogen assimilated by orchid and pine seedlings indicates that the net transfer of carbon was directed from pine to orchid seedlings, while the net transfer of nitrogen was channeled from green orchids to pine seedlings. *C*. *faberi* transferred 1.6 ± 0.3% of fixed carbon and 0.53 ± 0.07% of gained nitrogen to *P*. *yunnanensis* seedlings. Similarly, pine seedlings provided green-leaved *C*. *faberi* seedlings with about 3.0 ± 0.3% of fixed carbon and 0.68 ± 0.11% of acquired nitrogen (**Supplementary Figure S1**). Thus, a net carbon transfer was observed from pine to orchid seedlings in the latter association, but there was (almost) no net transfer of nitrogen between the two types of seedlings. In all mycorrhizal associations between *Cymbidium* and *P*. *yunnanensis* seedlings, a fraction of assimilated carbon and nitrogen was transferred via mycorrhizal networks to the shoots of the neighboring seedlings (**Supplementary Figure S1**). Regardless of whether the pathways were unidirectional or bidirectional, carbon transfer was always directed from pine seedlings to green orchids (**Figures 8a–c**). In contrast, nitrogen transfer was more differentiated and depended on orchid species: (i) unidirectional from orchid (*C*. *goeringii*) to pine seedlings (**Figure 8d**), (ii) bidirectional but almost no net transfer between orchid (*C*. *faberi*) and pine seedlings (**Figure 8e**), and (iii) bidirectional with a net transfer from orchid (*C*. *goeringii* var. *serratum*) to pine seedlings (**Figure 8f**).

## 4 Discussion

Despite the ecological importance of mycorrhizal networks, the mechanisms underlying carbon and nitrogen transfer between terrestrial orchids and co-occurring plant species remain poorly understood. In particular, systematic investigations into the directionality, magnitude, and drivers of these resource exchanges are still lacking. Our study, employing a simplified tripartite system and dual ^13^C and ^15^N labeling, provides direct evidence for bidirectional C and N transfer via a common *Ceratobasidium* fungus between *P*. *yunnanensis* seedlings and three *Cymbidium* orchids. Crucially, we found that the net flux and directionality of these transfers were not uniform but depended on the identity of the orchid species, revealing a previously underappreciated complexity in the functioning of common mycorrhizal networks.

Our observation that 1.0-3.7% of gained C and 0.20-12.2% of acquired N were transferred through the common mycorrhizal network aligns with the range reported in other systems (Simard and Durall, 2004). While numerous studies suggest transferred resources may be retained in fungal tissues (Waters and Borowicz, 1994; Watkins et al., 1996; Robinson and Fitter, 1999; Jakobsen and Hammer, 2015; Karst et al., 2023; Audisio et al., 2024), our data demonstrate that carbon and nitrogen were transferred through mycorrhizal networks to another plant in amounts comparable to those observed in ectomycorrhizal fungal networks (Simard et al., 2015). Additionally, a recent study in a temperate forest showed that a large amount of carbon can be traded between tall trees though ectomycorrhizal networks (Klein et al., 2016), but the net carbon exchange remained almost zero. The divergent transfer patterns among orchid species “unidirectional in *C*. *goeringii* versus bidirectional in *C*. *goeringii* var. *serratum* and *C*. *faberi*” may explain the longstanding controversies and inconsistencies in the literature regarding common mycorrhizal network mediated resource exchange (Robinson and Fitter, 1999; Karst et al., 2023). It suggests that generalized predictions based on source-sink theory alone are insufficient.

During long-term evolution, the three partners have developed a tripartite symbiosis in subtropical forests. *C*. *goeringii*, which grows in the forest understory in a light-limited environment, supposedly obtains carbon from pine trees, while pine trees obtain nitrogen from orchids via a common mycorrhizal network. When the tripartite symbiosis was established in the glasshouse, these species kept the same pattern of resource transfer and partitioning. In contrast, a bidirectional pathway between two of the *Cymbidium* orchid species and *P*. *yunnanensis* seedlings cannot be explained by the sink-source or the biological market theory (Noë and Hammerstein, 1995; Schwartz and Hoeksema, 1998). The mechanisms responsible for such bidirectional exchange need further investigations for better understanding of the function of mycorrhizal networks in forests. Nonetheless, the net carbon transfer in all mycorrhizal associations between the three *Cymbidium* orchids and *P*. *yunnanensis* seedlings was directed towards orchids. This could be ascribed to the mixotrophic character of the green-leaved *Cymbidium* orchids studied here (Motomura et al., 2010; Ogura-Tsujita et al., 2012). A previous study has demonstrated that low light levels lead to stronger mycoheterotrophy, while higher irradiances successively drive the orchids towards autotrophy (Preiss et al., 2010). In this study, the orchids were planted under high light levels in a glasshouse, but they still exhibited strong mycoheterotrophy. This indicates that the three *Cymbidium* orchid taxa exhibit strong, inherent mixotrophic traits compared to other orchid species engaged in tripartite symbioses. Another explanation is that their legacy of growing in the forest understory, in a light-limited environment could make them profit from photosynthetically fixed carbon originating from the coexisting pine trees, even under high light conditions. However, this needs further investigations.

The species-specific patterns are likely governed by a combination of factors. The fungus, isolated from *C*. *goeringii*, may have established a more specialized, efficient partnership with its original host, optimizing a unidirectional exchange (*i.e.* carbon transfer from pine to orchid and nitrogen transfer from orchid to pine). In contrast, its interaction with the other orchids might reflect a more generalist, balanced mutualism allowing for bidirectional flow. Furthermore, the strong inherent mixotrophy of these *Cymbidium* orchids (Motomura et al., 2010; Ogura-Tsujita et al., 2012), even under high light conditions, consistently created a carbon sink, explaining the universal net C transfer from pine to orchids. The differential N transfer (net, no net, or unidirectional) highlights how the nitrogen economy and demand can vary significantly even among closely related taxa, modulating the net outcome of the tripartite exchange. These carbon and nitrogen transfers between orchid and pine seedlings have confirmed the existence of several ectomycorrhizal pathways as predicted by Simard and Durall (2004), although a unidirectional pathway was not found for *C*. *goeringii* var. *serratum* and *C*. *faberi*. Such results indicate that the identity of orchid species that form mycorrhizal associations with fungi could modify the function of the mycorrhizal network in terms of resource transfer between the orchid and neighboring plants. This might well be the reason why so many observations on resource transfers in mycorrhizal networks are inconsistent or even contradictory (Jakobsen and Hammer, 2015; Simard et al., 2015).

These findings imply that common mycorrhizal networks are not merely passive pipelines but dynamic interfaces where the identity of the partner plants can fundamentally alter resource flow patterns. This plasticity could be a key mechanism enabling the coexistence of mixotrophic orchids in forest understories, allowing them to fine-tune their resource acquisition strategies. The gross simplification of the real-world complexity using two plant species linked by one fungal partner can provide highly valuable information regarding resource transfers between plants via mycorrhizal networks (Whitfield, 2007). Future research should move beyond binary plant-fungal systems to incorporate more complex networks involving multiple plant and fungal species to better mimic natural conditions and unravel the intricate web of belowground interactions. In conclusion, our study demonstrates that common mycorrhizal networks can indeed facilitate simultaneous bidirectional resource transfer, the magnitude and net outcome of which are controlled by the specific biological identity of the partners involved.

## Data Availability

The datasets presented in this study can be found in online repositories. The names of the repository/repositories and accession number(s) can be found in the article/supplementary material.
