# Supplementary material for: Mycorrhizal network: a bidirectional pathway between green-leaved terrestrial orchids and pine trees
Source: Front Plant Sci. 2025 Oct 28;16:1620153. doi: 10.3389/fpls.2025.1620153 (PMC12602444; doi:10.3389/fpls.2025.1620153)
Supplement: Supplementary file 1 [file Image1.docx]

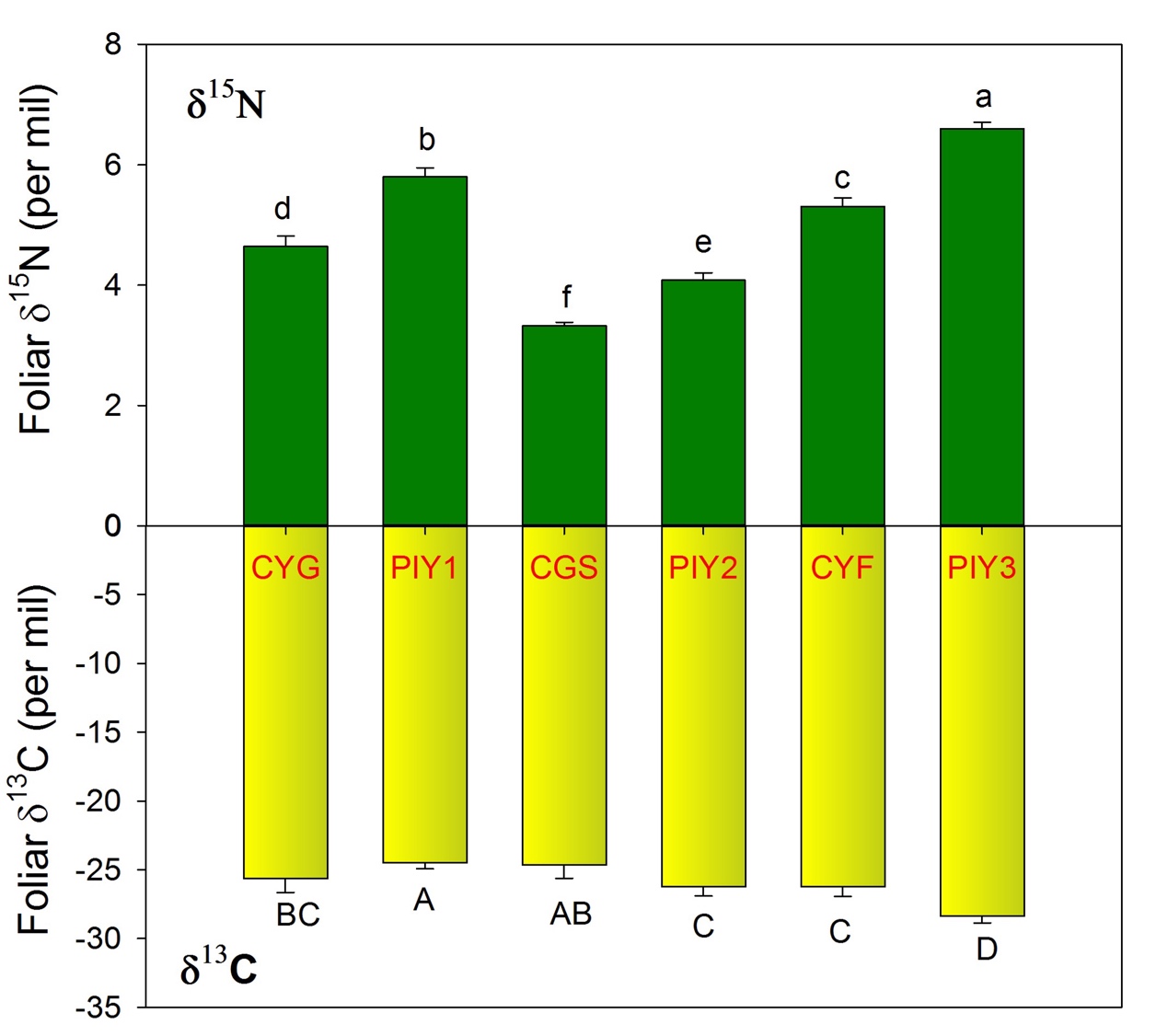


**Fig. S1** The natural abundance of carbon and nitrogen isotope in plants growing reference microcosms. The means ± SE of 4 replicates are presented. Different lowercase and uppercase letters indicate significant difference in foliar δ^15^N and δ^13^Cn, respectively, between orchid and pine seedlings at P=0.05 level. PIY - *Pinus yunnanensis*, CYG - *Cymbidium goeringii*, CGS - *C*. *goeringii* var. *serratum*, CYF - *C*. *faberi*.
